# Comprehensive pharmaceutical care to prevent drug-related readmissions of dependent-living elderly patients: a randomized controlled trial

**DOI:** 10.1186/s12877-018-0814-3

**Published:** 2018-06-04

**Authors:** R. Lenssen, K. Schmitz, C. Griesel, A. Heidenreich, J. B. Schulz, C. Trautwein, N. Marx, C. Fitzner, U. Jaehde, A. Eisert

**Affiliations:** 1 Hospital Pharmacy, University Hospital RWTH Aachen, Steinbergweg 20, 52074 Aachen, Germany; 2 Department of Urology, University Hospital RWTH Aachen, Pauwelsstr. 30, 52074 Aachen, Germany; 3 Department of Neurology, University Hospital RWTH Aachen, Pauwelsstr. 30, 52074 Aachen, Germany; 4 Department of Internal Medicine III, Gastroenterology, Metabolic Disorders and Internal Intensive Medicine, University Hospital RWTH Aachen, Pauwelsstr. 30, 52074 Aachen, Germany; 5 Department of Internal Medicine I, Cardiology, Pneumology, Angiology and Internal Intensive Medicine, University Hospital RWTH Aachen, Pauwelsstr. 30, 52074 Aachen, Germany; 6 Department of Medical Statistics, RWTH Aachen University, Pauwelsstr. 30, 52074 Aachen, Germany; 7 Institute of Pharmacy, Clinical Pharmacy, University of Bonn, An der Immenburg 4, 53121 Bonn, Germany; 8 Present address: Hospital Pharmacy, University Hospital Cologne, Kerpener Str. 62, 50937 Cologne, Germany; 9 Present address: Department of Urology, University Hospital Cologne, Kerpener Str. 62, 50937 Cologne, Germany

**Keywords:** Adverse drug reactions, Drug-related readmissions, Elderly patients, Pharmaceutical care

## Abstract

**Background:**

Elderly patients are vulnerable to adverse drug reactions (ADRs). Drug-related readmissions (DRRs) can be a major consequence of ADR. Therefore, this study aimed to investigate the effects of a ward-based, comprehensive pharmaceutical care service on the occurrence of DRRs as the endpoint in dependent-living elderly patients.

**Methods:**

A randomized, controlled trial was performed at a German University Hospital. Patients fulfilling the following criteria were eligible: admission to a cooperating ward, existing drug therapy at admission, 65 years of age and older, home-care or nursing home residents in ambulatory care, and a minimum hospital stay of three days. Patients received either standard care (control group) or pharmaceutical care (intervention group). Follow-up consultations were conducted for each patient at 1, 8, 26, and 52 weeks after discharge. The time to DRR was defined as the primary outcome measure and was analysed using the log-rank test. The Cox-proportional hazard model was used for risk factor analysis.

**Results:**

Sixty patients (*n* = 31 intervention group, *n* = 29 control group) participated in the study. For patients in the intervention group, the median time to DRR was prolonged; however, the level of statistical significance was not reached (log-rank test *P* = 0.068; HR = 3.28, *P* = 0.086). When the risk factors ‘age’ or ‘length of stay on the ward’ were added to the Cox proportional hazard model, patients in the control group exhibited a significantly higher risk of experiencing a DRR than patients of the intervention group (HR = 4.62; *P* = 0.028 including age and HR = 5.76; *P* = 0.033 including length of stay on the ward).

**Conclusions:**

Our findings demonstrate the successful implementation of ward-based, comprehensive pharmaceutical care for dependent-living elderly. Despite a low participation rate, which led to an underpowered study, the results provide a preliminary efficacy signal and effect size estimates to power a definitive trial.

**Trial registration:**

Clinicaltrials.gov identifier: NCT01578525, prospectively registered April 13, 2012.

## Background

Drug-related adverse events have been found to be the most common type of adverse events in hospitals and after discharge [1]. Moreover, up to 30% of all hospital admissions of elderly patients are drug-related [2, 3]. Therefore, strategies to prevent adverse drug reactions (ADRs) and drug-related hospital admissions are urgently needed, particularly for elderly patients [4–6]. In addition to other medication safety initiatives, national and international organizations recommend including pharmacists on health care teams to improve medication safety [7–11].

Several studies have demonstrated the positive patient-individual and economic impacts of pharmaceutical care [12–16]. Despite these results, few studies have evaluated the efficacy of pharmaceutical care services in hospitalized patients to reduce patient morbidity and mortality, particularly in high-quality and long follow-up trials [6]. Pharmaceutical care services are still limited and underrepresented in many countries in hospital as well as ambulatory care. The provision of those services, may prevent adverse drug reactions and subsequent hospital admissions. Therefore, the effect of a ward-based pharmaceutical care service on drug-related readmissions is a relevant and interesting topic for research [13, 17, 18].

Age and number of drugs have been found to be risk factors for ADR [19, 20]. Furthermore impaired cognition and a dependent-living situation are known risk factors [19, 21], which implies that elderly patients in dependent-living situations in ambulatory care, such as ‘being home-cared’ or ‘living in nursing homes’, are a vulnerable group for drug-related problems and ADRs. Moreover, those patient groups are often excluded in clinical trials, even in studies focusing on high-risk populations and elderly persons [7]. To the best of our knowledge, this study is the first to evaluate the effect of a ward-based, comprehensive pharmaceutical care service in dependent-living elderly patients and its potential to reduce the time until drug-related readmissions (DRRs).

## Methods

The study was set up as a randomized, controlled trial and conducted between April 2012 and March 2014, including a one-year recruitment and a one-year follow-up for each patient. The study was approved by the local ethics committee and is registered at clinicaltrials.gov.

### Participants

Patients 65 years of age and older, who were home-cared or nursing home residents in ambulatory care, admitted to one of the cooperating wards with a minimum hospital stay of expected three days and existing medication at admission were eligible for this study. Cooperation wards were four departments of non-intensive care units at the German University Hospital Aachen, Germany: the Departments of Urology, Neurology, Internal Medicine III (Gastroenterology and Metabolic Disorders) and Internal Medicine I (Cardiology, Pneumology and Angiology). We started recruitment with the three departments of the previous study (Urology, Neurology and Internal Medicine III). Because of the low participation rate, we added the department of Internal Medicine I after 6 months. Home-cared participants could receive care from informal or formal care-givers (e.g., family members or nurses). Informed consent was obtained from all individual participants or their legal representatives. After providing their consent, patients were randomized either to standard care (control group) or comprehensive pharmaceutical care (intervention group). Patients were randomized successively after being included in the study. Block randomization was chosen with 10 participants per block. A randomization list was generated at www.randomization.com. After study enrolment, the current number of included patient was the indicator for the allocation on the randomization list. Enrolment and allocation to the study group was conducted by the researcher (RL). The intervention was conducted by clinical pharmacists of the University hospital pharmacy (KS, CG, NH). Previous participation in the study was defined as the exclusion criterion.

### Intervention

The comprehensive pharmaceutical care service was established in a previous study and included a detailed medication history, medication reconciliation and a medication safety check directly after inclusion in the study and during the entire stay on the cooperating wards [20]. Medication was checked for new prescriptions, and a medication review was repeated with each newly prescribed drug. Medication safety checks included the plausibility of medication, check for drug allergies, renal/liver dysfunction and dosage adjustment, relevant laboratory data, contraindications, dosage, drug-drug interactions, adverse drug reactions, medications before surgery or other interventions, adequate duration of drug therapy, need for patient information, and therapeutic drug monitoring. Transitional care at discharge included medication reconciliation at discharge and providing recommendations for the discharge letter. After discharge, the comprehensive pharmaceutical care service ended, and all study patients received their ‘standard care’. The pharmacists had access to the data needed for the pharmaceutical care service. The service included also face-to-face counselling with the patients (e.g., for performing a detailed medication history or clarify potential problems in drug therapy).

Drug-related problems (DRPs) were identified during the entire pharmaceutical care process during the hospital stay. For each DRP, a specific recommendation was addressed to the health care team. Each recommendation was discussed with the healthcare team, and the physician decided if he or she followed the recommendations. The medication was checked again and registered, if the recommendation was partly or fully followed. The DRPs were documented and classified using the APS-Doc system [22], which we extended with three more subcategories in ‘others’.

All pharmaceutical care activities were defined in standard operating procedures and performed by independent, trained clinical pharmacists to ensure the same standard of intervention for all patients in the intervention group. To ensure the equality of the observations for both treatment groups, intervention pharmacists (CG, KS, NH) worked independently from the researcher (RL). The intervention pharmacists were trained in all details of the SOP. They had one to three years of experience in clinical pharmacy. The intervention was established in three departments for the duration of the previous study. After closing that study, the intervention was not transferred to daily routine. Thus, standard care did not include clinical pharmacists in the health care team on the ward. Clinical pharmacists in this hospital are working in the hospital pharmacy and have contact with the wards mainly by telephone. A detailed medication review was only performed on explicit request of a physician. As described in our previous study, the routine medication process on the ward was performed by physicians and nurses.

### Data collection

For the control and intervention groups, a researcher collected all relevant information on case report forms (CRF), designed for this study, including the current medication on the ward and demographic data, such as age, gender, social status (home-cared, nursing home resident), laboratory data and diagnoses. During the entire stay on the ward, ADR-suspicious symptoms were detected and discussed with the physician if necessary.

After discharge from the cooperating wards patients were contacted as pre-defined in the study protocol 1, 8, 26 and 52 weeks after discharge. Follow-up visits were conducted as semi-structured interviews, preferably with an interview guide, with the patient or responsible relatives or nurses who cared for the patient. Within the interviews current medication, ADR-suspicious symptoms and hospital readmissions to any hospital that occurred during the time after the last interview were documented. Additionally, available laboratory data, discharge letters and other patient documentation were examined.

Medication changes were determined from discharge medication to medication of the follow-up point. Changes in the strength, dose, dosage, dosing regimen or drug were included.

### Sample size estimation

Based on the previous results of Gillespie et al., an 80% reduction in drug-related readmissions was assumed. Previous own results on the cooperating wards showed 2.6 DRPs per patient 65 years and older [20]. This DRP rate was consistent with the results of Gillespie et al. (2.6 DRPs/patient), despite their older population (80 years and older) [13]. In addition, the following parameters were set: drop-out rate 25%, power 80%, significance level α 0.05, and two-sided log rank test. The recruitment period was set at 12 months, with a follow-up period of 12 months for each patient. A sample size of 278 patients (139 patients and 15 events per group) was calculated using nQuery Advisor® 7.0, Statistical Solutions Ltd., Cork, Ireland. Therefore, the sample size was set to 300 patients.

### Outcome measures

The primary endpoint was the occurrence of drug-related readmissions (DRRs), measured over one year at four pre-defined contact times after discharge. DRR was defined as re-hospitalization of a discharged patient due to an adverse drug reaction (ADR) in any hospital. ADR-suspicious symptoms were defined as symptoms occurring in plausible context of medication use including inherent ADRs and ADRs due to medication errors. ADR-suspicious symptoms also included changes in laboratory data in a clinically relevant manner. All ADR-suspicious symptoms reported at the follow-up consultation were documented. It was investigated and documented when the symptoms occurred.

All ADR-suspicious symptoms and hospital readmissions were reviewed in a two-step process after data collection period. First, three pharmacists assessed all cases of ADR-suspicious symptoms and hospital readmissions (*n* = 157); they were blinded regarding the allocation of the patients to the control or intervention group. The ADR-suspicious symptoms were classified as ‘no ADR’, ‘potential ADR’ or ‘ADR’ using the causality criteria by Arimone et al. [23]. In a second step, all ‘potential ADR’ (*n* = 34) were again assessed by three independent experts (community pharmacist, hospital pharmacist, physician). A majority decision was made after the experts voted on a scale as described by Arimone et al. [23]. Potential ADRs were thereby classified in seven causality levels: ‘ruled out’, ‘unlikely’, ‘doubtful’, ‘indeterminate’, ‘plausible’, ‘likely’, ‘certain’. The causality levels ‘certain’, ‘likely’ and ‘plausible’ were regarded as ADR. Preventability and ameliorability were assessed according to Schumock and Thornton [24]. All preventable/ameliorable ADRs were categorized, according to their severity using the NCC-MERP criteria [25].

In our study, ADR was defined as a harmful and unintended reaction on a medical product, which can occur under conditions of intended use or due to a medication error [26].

Moreover, as secondary endpoints, adverse drug reactions, potentially inappropriate medication (PIM) using the PRISCUS list [27] and the number of changes in medication after discharge were documented. In the intervention group, the number of drug-related problems and the number of accepted recommendations were assessed as further endpoints.

A drug-related problem (DRP) was defined as an “event or circumstance involving drug therapy that actually or potentially interferes with desired health outcomes”, as stated by the Pharmaceutical Care Network Europe [28].

### Data analysis

Data from CRFs were entered in Microsoft Excel® (Microsoft Corporation, Redmond, WA, USA) spreadsheets for analysis. Statistical analysis was performed using SAS® 9.1.3 (SAS Institute Inc., Cary, NC, USA). DRRs were analysed using the log-rank test to compare the time until occurrence of DRR. To consider potential risk factors on DRR Cox proportional hazard models were generated including ‘treatment group’ and one of the above-mentioned risk factors as covariates. The risk factors ‘gender’ and ‘living situation’ were included as binary variables, all other risk factors (‘age’, ‘length of stay on the ward’, ‘number of PIM drugs’, ‘number of changes in medication after discharge’, ‘number of drugs during stay on the ward’) were included as continuous variables. Hazard ratios (HR) were calculated to express the risk ratio for DRRs between the treatment groups [29].

To compare the baseline data in both study groups we used Satterthwaite t-test for ‘age’, length of hospital stay’ and ‘number of drugs during hospital stay’. For the parameters ‘gender’ and ‘living situation’, we used the Fisher exact test.

## Results

**Fig. 1:**
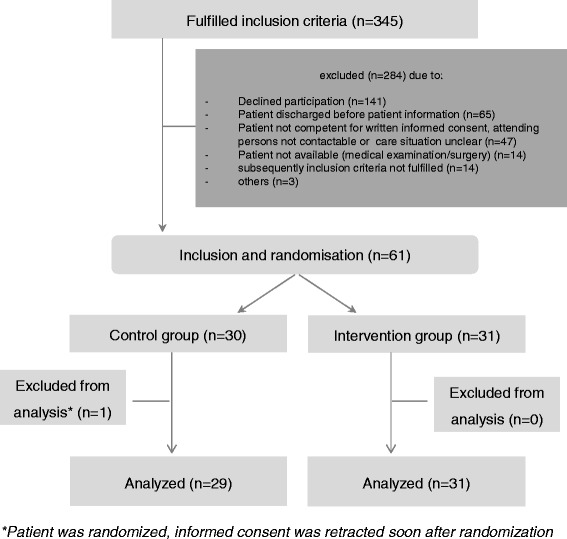
Patient flow chart

During the one-year recruitment period, 61 patients were eligible and willing to participate (participation rate 17.7%). The number of recruited patients in each department is shown in Appendix 1. Thirty patients were allocated to the control group and 31 patients to the intervention group. One patient in the control group wished to be excluded from the study soon after randomization. Hence, 60 patients were analysed (for the study flow chart, see Fig. 1) after the previously defined one-year follow-up for each patient ended. Forty patients completed the follow-up. The inability to reach a patient (e.g., the patient moved to new address or died) caused censoring. The details are listed in Appendix 2.

The mean age of the participants was 77.6 ± 7.9 years; 60% of the participants were female, and 70% of participants were home-cared. On average, the participants had 16.8 ± 6.9 drugs in their medication on the wards (see Table 1). Five patients received no pharmaceutical care consultation because they were discharged before counselling. We performed an intention-to-treat analysis and included these data in the analysis.

**Table 1:** Summary of patient characteristics and endpoints in control and intervention group

	Item	Control group (*n* = 29)	Intervention group (*n* = 31)	*p*-Value
Patient characteristics	Gender (female, %)	17 (58.6%)	19 (61.3%)	1.000
	Living situation (home-cared, %)	22 (75%)	20 (64.5%)	0.4051
	Age (mean, SD, range; years)	79.5 ± 8.62 (66–99)	75.9 ± 6.87 (66–91)	0.0856
	Length of stay on ward (mean, SD, range; days)	10.1 ± 6.0 (3–29)	11.9 ± 8.5 (4–40)	0.0671
	Number of drugs during hospital stay (mean, SD, range)	16.3 ± 6.3 (7–32)	17.3 ± 7.3 (6–40)	0.5649
Drug-related readmissions (DRRs)	Number	7	6	
	Number of patients with DRR	7	3	
Adverse drug reactions (ADRs) during follow-up	Number	14	21	
	Number of preventable/ameliorable ADR (NCC-MERP category F-H) regarded as “preventable DRR”	4	1	
	Number of preventable/ameliorable ADR (NCC-MERP category D-E)	2	7	
Adverse drug reactions (ADRs) during hospital stay	Number	9	9	
Potential inappropriate medication (PIM)	One-year prevalence of PIM	20	31	
Changes in medication after discharge during follow-up	Number per patient	16 ± 11 (3–47)	16 ± 10 (3–52)	

*SD* Standard deviation

Of the 30 patients in the intervention group, 26 received pharmaceutical care (see Fig. 1), and 100 DRPs were detected. Of them, 72% were partly or fully solved by the healthcare team in cooperation with the pharmacists. The detailed APS-Doc categories are shown in Appendix 3.

The mean time for providing pharmaceutical care during the initial hospital stay was 5.9 h per patient.

### Time to drug-related readmission

**Fig. 2:**
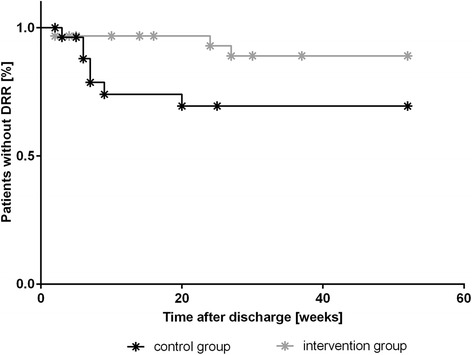
Kaplan-Meier plots for the time-dependent occurrence of drug-related readmissions (DRR). Censored data are marked as *

Regarding the one-year follow-up in total, the median time until onset of DRR in the intervention group was longer compared to the control group. In the log-rank test, however, this difference was not significant (*p* = 0.068, see Fig. 2). Characteristics of the curves showed rare and later DRRs in the intervention group than in the control care group. The risk for DRR was higher under standard care compared to comprehensive pharmaceutical care (HR: 3.276 [CI: 0.844–12.716]; *p* = 0.0864). However, no statistical significance was shown.

### Preventable drug-related readmissions

In total, thirteen DRRs were documented during the 12-month follow-up (see Table 1). Seven patients in the control group (CG) and three patients in the intervention group (IG) exhibited at least one DRR in the follow-up period (see Table 1).

Thirty-five ADRs were observed during follow-up period. 14 ADRs (40%) were judged to be preventable or ameliorable and categorized according to NCC-MERP for severity. Preventable or ameliorable ADRs of category F-H requiring a hospital stay can be regarded as preventable DRRs. Four preventable DRRs occurred in the control group and one in the intervention group (see Table 1).

Among the 60 patients, we measured 52 readmissions to any hospital during the follow-up period. Of them, 13 readmissions were judged to be drug-related readmissions in 10 patients, as shown in Table 1. These drug-related readmissions (*n* = 13) caused a mean stay of 19.8 ± 19.1 days. Preventable DRRs (*n* = 5) caused a mean stay of 13.4 ± 30.7 days.

### Risk factors for drug-related readmissions

A detailed risk factor analysis was performed based on Cox proportional hazard models, including the potential covariates age, gender, living situation, length of stay on the ward, number of drugs on the ward, number of PIM using the PRISCUS list [27] and number of changes in medication after discharge (see Table 2). Age, the length of stay on the ward, and the number of changes in medication after discharge were found to be statistically significant risk factors for DRR.

**Table 2:** Covariate analysis in a Cox-proportional hazard model. Univariate analysis was performed for each covariate

Covariate	Effect risk factor on DRR		Effect treatment group on DRR	
	HR_RF_ [CI]	*P* value	HR_TG_ [CI]	*P* value
Age [years]	0.86 [0.76–0.97]	0.014*	4.62 [1.18–18.13]	0.028*
Gender	0.81 [0.23–2.79]	0.732	3.26 [0.84–12.66]	0.088
Living situation	1.01 [0.21–4.94]	0.989	3.28 [0.82–13.09]	0.092
Length of stay on ward [d]	1.10 [1.01–1.19]	0.020*	5.76 [1.15–28.85]	0.033*
Number of PIM drugs	0.98 [0.34–2.80]	0.966	3.26 [0.83–12.81]	0.090
Number of changes in medication after discharge	1.06 [1.01–1.11]	0.013*	2.51 [0.62–10.17]	0.196
Number of drugs (during stay on the ward)	1.04 [0.95–1.14]	0.387	3.54 [0.90–13.89]	0.070

The hazard ratio (HR) describes the influence of the risk factor on the risk for DRR (“effect risk factor”) or the risk for a DRR in the control group compared to the intervention group (“effect treatment group”)

*RF* risk factor, *TG* treatment group, *CI* confidence interval, *PIM* potentially inadequate medication

*P < 0.05

An older age was associated with a lower risk of DRR (HR = 0.86; CI: 0.76–0.97; *P* < 0.05). Including age in the univariate Cox proportional hazard model, the control group showed an approximately five-fold risk for DRR compared to the intervention group (HR = 4.62; CI: 1.18–18.13; *P* < 0.05).

Each day the hospital stay was prolonged, the risk of a DRR increased by 1.1-fold (HR = 1.10; CI: 1.01–1.19; *P* < 0.05). A longer stay of one week resulted in a two-fold risk increase (HR = 1.91; CI:1.11–3.3; *P* < 0.05). Including this risk factor in the univariate model, the control group had an approximately six-fold risk for DRR compared to the intervention group (HR = 5.76; CI: 1.15–28.85; *P* < 0.05).

Although the number of changes in medication was found as a risk factor on DRR, the treatment group had no significant effect on DRR when including this risk factor.

For the time to ADR during follow-up in the intervention group compared with the control group, the log-rank test (*p* = 0.0684) and hazard ratio (HR:0.305; CI: 0.079–1.185; *p* = 0.0864) showed no statistical significance.

## Discussion

This study showed a potential positive impact of a comprehensive pharmaceutical care service and its potential to increase the time until DRR after discharge of elderly, cared patients in dependent-living situations. In the control group, there was a tendency for a higher risk for DRR compared to the intervention group receiving pharmaceutical care. However, the hazard ratio did not reach statistical significance, likely due to the low number of events.

The low participation rate of 17.7% must be considered when interpreting the results. The possible reasons for the non-participation were high age and morbidity of the patients. In our study, 41% of the patients declined to participate. Another study, with a decline rate of 36%, showed similar factors (e.g., age, gender, morbidity) for non-participation in ambulatory care [30]. Home-care patients and nursing home residents are often excluded from other studies. In our study, 14% of the potential participants were not able to give written informed consent themselves. In addition, no legal representative was available. These circumstances make it more difficult to conduct randomized-controlled trials in this patient group. Approximately 20% of the possible participants were discharged before they could be informed about the study.

To obtain the most reliable results from this study group, a risk factor analysis was performed. In the risk factor analysis, age, the length of hospital stay and the number of changes in medication after discharge were found to have significant influence on DRRs, which is in accordance with other studies for ADRs and drug-related hospital admissions [19, 31]. When adding age or the length of stay to the Cox proportional hazard analysis, the effect of pharmaceutical care was statistically significant. Thus, this analysis underlines the results of previous studies in patients with long hospital stays [21, 32]. In contrast to some studies, the younger patients were more vulnerable for DRR than the older patients in this patient population [19, 20]. Some other studies did not identify ‘age’ as a risk factor for ADRs and DRRs [21, 32–34].

A positive impact on preventable ADRs and medication-related emergency department visits, medication-related hospital admissions and readmissions in different patient populations has also been reported in previous studies [12–14, 35]. This current study is the first to suggest a positive effect of pharmaceutical care for dependent-living elderly in particular. Moreover, the results suggest a time-dependent effect of ward-based pharmaceutical care services. After discharge, the patients had the greatest benefit from the previously performed ward-based pharmaceutical care during the first ten weeks. It seems reasonable that ward-based pharmaceutical care can influence the first weeks after discharge at most, which implies that pharmaceutical care services are also needed in ambulatory care to optimize medication of those patients continuously and to prevent ADRs and DRRs. In our study, we detected 35 ADRs in 60 patients. Thirteen of these ADRs led to drug-related readmissions in ten patients, causing a mean hospital stay of 19.8 days in this group. One patient was readmitted four times due to ADRs. Fourteen ADRs were regarded as ameliorable or preventable. Five of them caused hospital stays averaging 13.4 days. From an economic perspective, these costs are preventable for the health care system. Thus, effective methods to reduce ADRs and DRRs are needed to address the patients’ and health-care system needs.

The absolute number of ADR was higher in the intervention group than in the control group. Perhaps, the intensive medication review by the pharmacist gave more signals for the researcher to detect ADR-suspicious symptoms.

Due to the limited number of participants, the efficacy of the intervention could not be confirmed in this study. Nevertheless, the results show the potential of pharmaceutical care to influence patient-relevant endpoints and provide the basis to plan future studies in a more feasible manner, including our experience that multimorbidity and health restrictions may compromise the participation rate in the target population.

### Strengths

The study was designed as randomized-controlled trial with a long follow-up of 12 months to address the need of high-quality and long follow-up trials, as stated in a Cochrane Review [6]. Parallel-group design was preferred to minimize a possible time-dependent bias. To ensure equality of observation for both treatment groups, the intervention pharmacists (CG, KS, NH) worked independently from the researcher (RL).

The results of this study show the potential efficacy of the intervention in a daily practice setting. The intervention was conducted as intended by the study protocol. The intervention pharmacists found 2.6 DRPs per patient on average, which aligns with the results of our previous study [20]. However, five patients received no intervention because their discharge occurred in such a narrow time frame that the intervention could not be performed.

### Limitations

The small number of participants resulted in an underpowered study. Thus, statistical significance could only be shown after including covariates in the model.

As in many other studies, the Hawthorne effect could have influenced the behaviour of physicians, nurses, patients and relatives. The long follow-up may have induced a recall bias, as patients or contact persons might not have remembered less severe events that happened long ago. Additionally, a detection bias may have influenced the results, as the follow-up interviews were not blinded. We tried to avoid this bias using standardized questions. Furthermore, there may be a risk of subjectivity in the outcome assessment; this risk was minimized by having more than one reviewer.

Our study may have limited generalizability to non-academic hospitals and other university hospitals because of the mono-centric study design, the selection of cooperating wards and the low participation rate. In contrast to other studies, however, we included patients of different medical disciplines representing a broader patient population [12–14].

All patients had different general practitioners, physicians, community pharmacists and other healthcare professionals who were involved in therapy and might have influenced their medication or their risk for rehospitalization. In future studies, these confounders could be documented in a structured form and might be added to the risk factor analysis.

## Conclusions

This study shows the potential of a comprehensive pharmaceutical care service to reduce DRRs after discharge for elderly, cared patients in dependent-living situations. Thus, the results suggest a preliminary efficacy signal for the intervention and provide effect size estimates for powering definitive trials of this intervention in a larger patient population.

## Appendix 1

**Table 3:** Number of participating patients in each department

Department	Control group	Intervention group
Department of Urology	2	2
Department of Neurology	10	18
Department of Internal Medicine III	15	8
Department of Internal Medicine	2	3
Total	29	31

## Appendix 2

**Table 4:** Censoring during the study follow-up

Time of contact	Number of patients in total	Number of censoring in control group	Number of censoring in intervention group
At discharge	60	0	0
1 week after discharge	60	0	0
8 weeksafter discharge	51	7	2
26 weeks after discharge	45	3	3
52 weeks after discharge	40	1	4

## Appendix 3

**Table 5:** DRP categories of the intervention group (n = 26 patients)

DRP category	Number of DRP
Drug	10
Dosage form/drug strength	1
Dosage	7
Indication	14
Contraindication	3
Drug-drug interaction	36
Adverse drug reaction	5
Administration/adherence	5
Administration	5
Other	14
Total	100

## Abbreviations

ADR: Adverse drug reaction
CG: Control group
CRF: Case report form
DRP: Drug-related problem
DRR: Drug-related readmission
HR: Hazard ratio
IG: Intervention group
PIM: Potential inappropriate medication
